# A Clinical Study of Anaerobic Wound Infection

**Published:** 1917-12

**Authors:** 


***A Clinical Study of Anaerobic Wound Infection.*** (Analysis of 107 cases of Gas Gangrene.) Abs. from the *Lancet*, Dec. 23, 1916. p. 1058. By M. H. F. IVENS, Médecin-chef Hôpital Auxiliaire 301.

I. observed 464 cases of gas infection, of which 107 were clinically gas gangrene.

She emphasizes the importance of complete bacteriological study and of the careful examination of X-ray plates, which in most cases show the situation of gas bubbles or streaks.

The author notes as important factors in the production of gaseous gangrene :

1. The proximity of contaminated soil : wounds of the lower limbs showed a mortality three times as great as those of the upper, though wounds of the upper were more frequent. 2. Shell wounds were six times as frequent in gas gangrene as in ordinary infected wounds. 3. The presence of an infected wad of clothing kept up infection. 4. The interval between the wound and the first surgical intervention : insignificant wounds might cause fatal results if untreated and severely infected. 5. Early treatment was most important in the prevention of gas gangrene. 6. Vascular lesions were an important factor when due to injury; as a remedial measure, such as the ligature of great vessels, they were not important; 22 cases with vascular lesions were followed by gangrene in six only. 7. 60 o/o of gas infected cases had fractures, and 71 o/o of those of gas gangrene. 8. Wounds of the calf, trunk, or hip joint were specially dangerous if deeply seated. 9. Tissue injury had an important influence; gas abscesses were frequently seen in gas infections at the site of subcutaneous injections or near simple fractures in the same case. 10. Intramuscular tension from within or without was a potent aid in the production of gangrene. 11. Joint injuries occurred in 13 o/o of gas infections, and in 20 o/o of gas gangrene. They increased the gravity of cases, and the damaged parts were difficult to immobilize without pressure.

There was usually a mixed bacterial flora : B. perfringens (aerogenes capsulatus) was present in nearly every case, B sporogenes in 41 cases, Vibrion septique in 6 cases (several fatal), B. histolyticus B. Hibler IX, and B. œdematiens were all reported, but less frequently. Streptococci of a virulent type were present in 59 cases and added to the gravity of the infection. Tetanus occured in 15 cases and was demonstrated bacteriologically in 7. Intrathecal administration of anti-tetanus serum, 30 cc., at a dose, together with subcutaneous injections up to 30 and 40 cc. a day proved successful.

I. noted seven clinical varieties of gas gangrene, which she classified as follows : (1) Classic, (2) toxic or edematous, (3) mixed forms, (4) local gas abscess, (5) superficial and deep seated gas phlegmon, (6) chronic and latent infections, (7) gas septicemia or pyemia.

Of 464 cases of gas infection 42 were fatal, 25 dying from the gas gangrene without complications.

Amputations were considered necessary in advanced cases; 65 were performed by the open method with lateral incisions : there were 48 recoveries.

When gangrene was limited to groups of muscles or joints, excisions were performed 41 times: there were 33 recoveries.

Hypertonic salt solution used alone was unsuccessful, but combined with 2 1/2 o/o carbolic acid gave good results. Iodine vaccine-therapy appeared to be more successful when there was also streptococcal infection. Other methods were used, such as continuous irrigation with eusol, Carrel solution, or normal saline (all apparently giving the same results).

Ten cases of very severe gas gangrene had also been treated by antiperfringens, anti-œdematiens, and anti-vibrion septique serum (provided by Dr. Weinberg, see p. 128 f.). In 5 cases, one being a septicemia with triple anaerobic infection, the treatment was successful. The fatal cases were already septicemic before the serum was administered, but there was evidence of beneficial effect.

